# Supplementary material for: Neural correlates of obesity across the lifespan
Source: Commun Biol. 2024 May 28;7:656. doi: 10.1038/s42003-024-06361-9 (PMC11133431; doi:10.1038/s42003-024-06361-9)
Supplement: Supplementary file 2 — Supplementary Information [file 42003_2024_6361_MOESM2_ESM.pdf]

Supplementary Figures

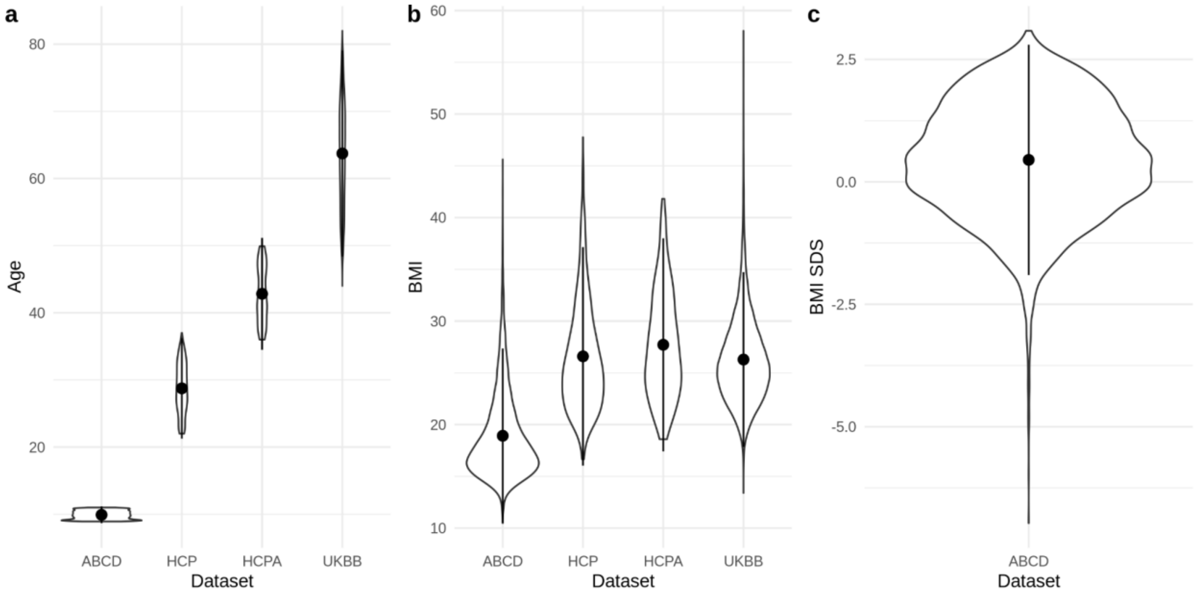

Figure S1 - age and BMI distribution of each sample included in the study

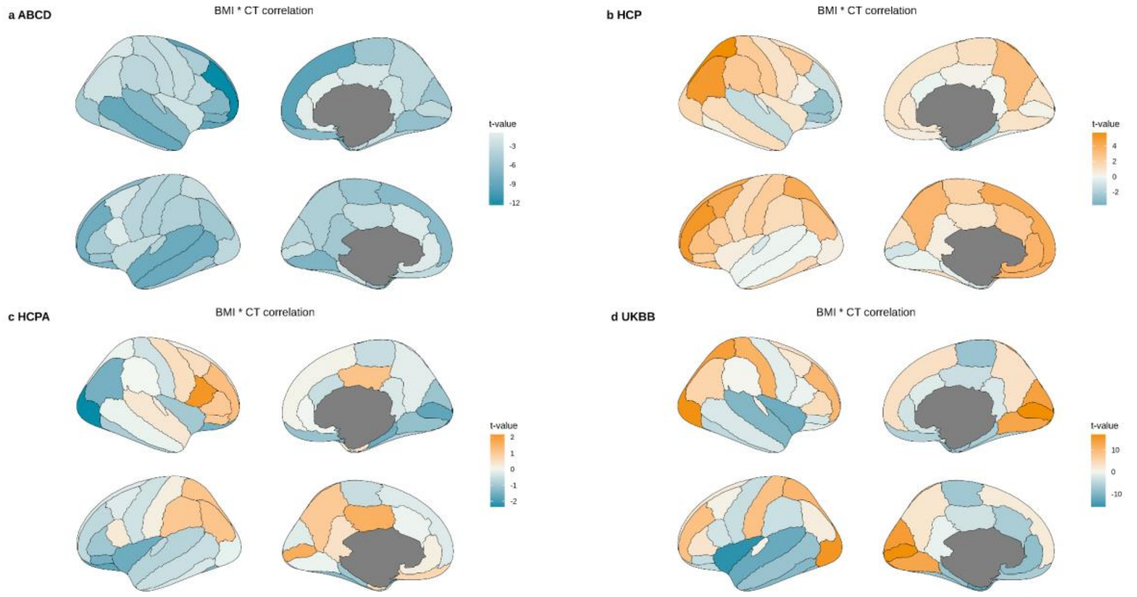

Figure S2 - Relationship between body mass index (BMI) and cortical thickness in 4 samples tested without regressing out effects of socioeconomic status.

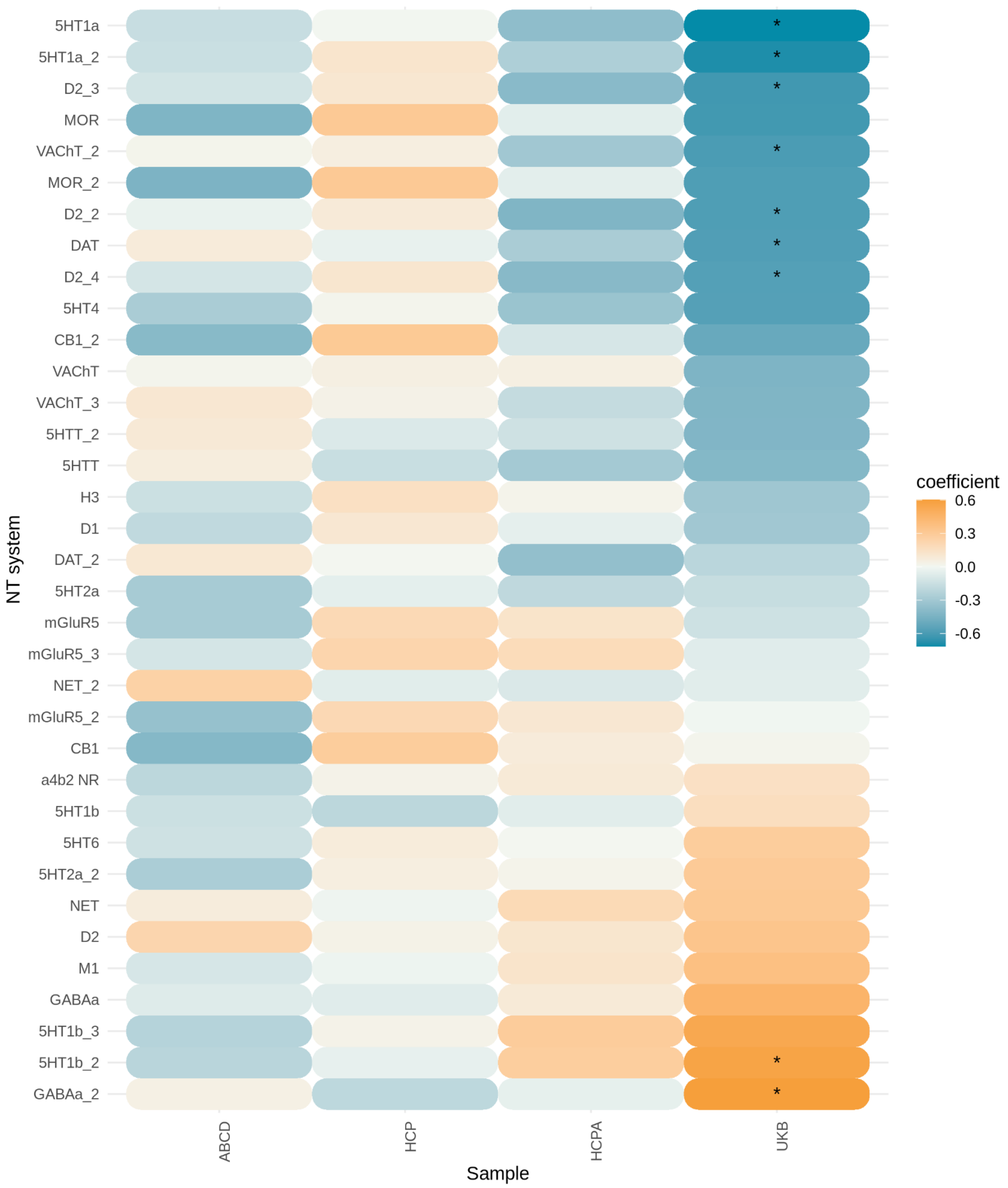

Figure S3 - correlations between all available neurotransmitter maps and obesity maps. Refer to Table S5 for details on studies used to derive neurotransmitter maps. * denotes significant associations. BMI - body mass index. CT - cortical thickness. GABAa - gamma-aminobutyric acid receptor a. 5HT1b - serotonin 1b receptor. M1 - muscarinic M1 receptor. NET - norepinephrine transporter. 5HT6 - serotonin 6 receptor. a4b2 NR - α4β2 nicotinic receptor. mGluR5 - metabotropic glutamate receptor 5. 5HT2a - serotonin 2a receptor. D1 - dopamine D1 receptor. H3 - histamine H3 receptor. 5HTT - serotonin transporter. VAChT - vesicular acetylcholine transporter. CB1 - cannabinoid receptor 1. 5HT4 - serotonin 4 receptor. DAT - dopamine transporter. MOR - μ-opioid receptor. D2 - dopamine D2 receptor. 5HT1a - serotonin 1a receptor.

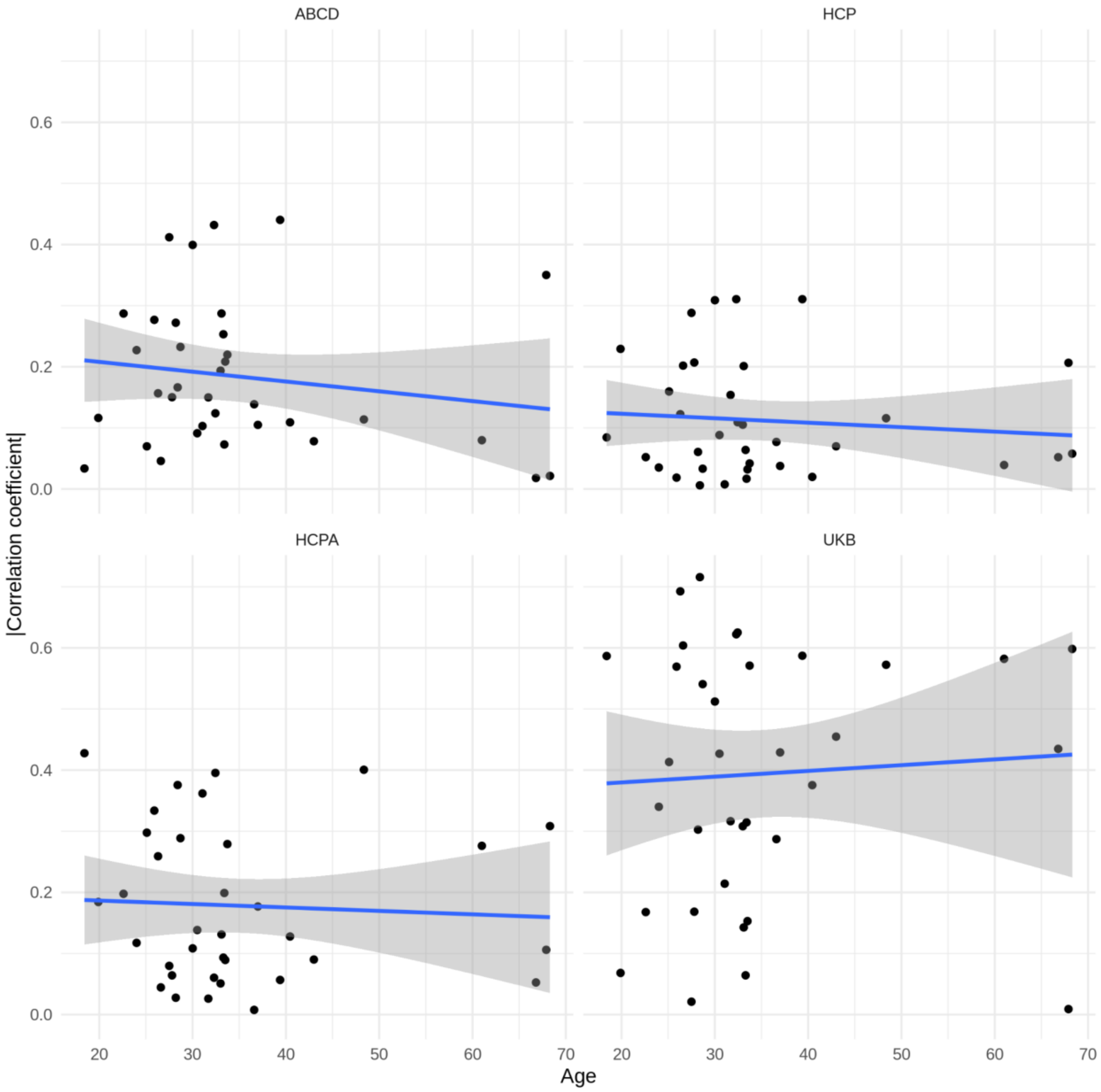

Figure S4 - relationship between correlation coefficient in the neurotransmitter analyses (correlation between obesity brain maps and neurotransmitter availability maps) and age of the sample from which the neurotransmitter maps were derived. There was no significant relationship (p>0.05) indicating that age of neurotransmitter sample did not affect correlation between obesity maps and neurotransmitter availability. Shaded area depicts confidence intervals.
