# Supplementary material for: Neural correlates of obesity across the lifespan
Source: Commun Biol. 2024 May 28;7:656. doi: 10.1038/s42003-024-06361-9 (PMC11133431; doi:10.1038/s42003-024-06361-9)
Supplement: Supplementary file 3 — Description of Supplementary Data File [file 42003_2024_6361_MOESM3_ESM.pdf]

Description of Additional Supplementary Materials

File name: Supplementary Data 1

Description: Associations between cortical thickness and BMI in the ABCD sample

File name: Supplementary Data 2

Description: Associations between cortical thickness and BMI in the HCP sample

File name: Supplementary Data 3

Description: Associations between cortical thickness and BMI in the HCP Aging sample

File name: Supplementary Data 4

Description: Associations between cortical thickness and BMI in the UKBB sample

File name: Supplementary Data 5

Description: Associations between neurotransmitter systems and cortical thickness changes in obesity in all samples
